# What factors are most important for the development of the maternal–fetal relationship? A prospective study among pregnant women in Danish general practice

**DOI:** 10.1186/s40359-020-00499-x

**Published:** 2021-01-04

**Authors:** Ruth K. Ertmann, Christine W. Bang, Margit Kriegbaum, Mette S. Væver, Jakob Kragstrup, Volkert Siersma, Philip Wilson, Melissa C. Lutterodt, Johanne Smith-Nielsen

**Affiliations:** 1 The Research Unit for General Practice and Section of General Practice, Department of Public Health, University of Copenhagen, Copenhagen, Denmark; 2 Departmant of Psychology, University of Copenhagen, Copenhagen, Denmark; 3 Centre for Rural Health, Institute of Applied Health Sciences, University of Aberdeen, Aberdeen, Scotland UK

**Keywords:** Maternal antenatal attachment, Pregnant, General practice, Cohort, Risk factors, Pregnancy-related symptoms

## Abstract

**Background:**

Development of the maternal antenatal attachment (MAA) constitutes an important aspect of the transition into motherhood. Early identification of women at risk of developing a poor MAA provides possibilities for preventive interventions targeting maternal mental health and the emerging mother-infant relationship. In this study, we investigate the relative importance of an extensive set of psychosocial, pregnancy-related, and physiological factors measured in the first trimester of pregnancy for MAA measured in third trimester.

**Methods:**

A prospective study was conducted among pregnant women in Danish general practice (GP). Data were obtained in the first and the third trimester from pregnancy health records and electronic questionnaires associated with routine GP antenatal care visits. The Maternal Antenatal Attachment Scale (MAAS) was used to assess maternal antenatal attachment. The relative importance of potential determinants of maternal antenatal attachment was assessed by the relative contribution of each factor to the fit (R^2^) calculated from multivariable regression models.

**Results:**

The sample consisted of 1328 women. Low antenatal attachment (Total MAAS ≤ 75) was observed for 513 (38.6%) women. Perceived social support (having someone to talk to and having access to practical help when needed) emerged as the most important determinant. Furthermore, scores on the MAAS decreased with worse self-rated health, poor physical fitness, depression, increasing age, having given birth previously, and higher education.

**Conclusion:**

Pregnant women reporting lack of social support and general low physical and mental well-being early in pregnancy may be at risk for developing a poor MAA. An approach targeting both psychosocial and physiological well-being may positively influence expectant mothers’ successful adaptation to motherhood.

## Background

The pregnant woman’s successful adaptation to motherhood is closely linked with the development of an emotional bond with the fetus, that is, the mental representation of the fetus and feelings of being connected with it [1–3]. In this study, we investigate which routinely collected factors should be considered if one wishes to assess the pregnant woman’s relationship with the fetus.

While authors have used several terms to capture this phenomenon, for example, ‘antenatal attachment’ [4], ‘maternal–fetal attachment’ [5], and ‘prenatal attachment’[6], in the present paper, following Condon (1993), we use the term maternal antenatal attachment (MAA). In 1981 MAA was defined by Cranley as “the extent to which women engage in behaviours that represent an affiliation and interaction with their unborn child” [7]; it is characterised by cognitions, feelings and behaviours towards the fetus [8], and intensifies as pregnancy progresses [9]. Stressing the representational aspects of the phenomenon, Condon (1993) described MAA as the emotional bond that the pregnant woman develops with the fetus [4]. While the quality of attachment is usually described as secure/insecure (e.g. [10, 11], MAA is typically described in terms of high/low ‘intensity’, reflecting strength of the relationship, and high/low ‘quality’ (reflecting positive/negative feelings towards the fetus (e.g. [12]).

MAA is important because it relates to a range of indicators of the quality of the postnatal mother-infant relationship, such as self-reported bonding with the infant [13, 14], maternal mind-mindedness throughout the first two postnatal years [15], and a meta-analysis reports modest but robust associations between MAA and the quality of mother-infant interaction [16]; Mother-infant interaction quality is documented to predict mother-infant attachment [17] which in turn predicts children’s socio-emotional and mental health outcomes [18–20]. Finally, low scores on measures of MAA are consistently found to be associated with perinatal depression and anxiety [9, 13, 14] which are risk factors for adverse child cognitive and behavioural outcomes [21] and long-term mental health problems [22].

A meta-analysis of 72 studies [9] attempted to assess the relative predictive properties of a range of demographic, psychosocial and pregnancy-related factors associated with MAA. However, since most of the included studies used samples smaller than 100, and as there was substantial heterogeneity in effect sizes, the authors concluded that more research on the subject was needed. Similarly, two reviews stress the relationship between MAA, and psychosocial and pregnancy-related factors, yet both call for future research in larger, more diverse samples [23, 24].

While the existing research supports the view that psychosocial factors, and in particular social support, predict MAA, to our knowledge, no study has investigated whether pregnant women’s physical symptoms, pregnancy-related discomfort and sleep problems also play a role. In the present study, we add to the literature by integrating these factors into our analysis of findings from a large cohort of pregnant women. These additional factors may influence MAA directly or indirectly through psychological distress, and/or through endocrine mechanisms. In particular, overall well-being—physical as well as mental—may be important for optimal transition to motherhood and the maternal sense of connection with the fetus [25].

Here we investigate the relative contribution of an extensive set of first trimester factors either found in the woman’s pregnancy record or which are easily examined during a first pregnancy consultation to the strength of MAA measured in the third trimester using the Maternal Antenatal Attachment Scale (MAAS) [4]. Knowledge of the factors—in particular those that are present and can be identified early in pregnancy—that are most important for a healthy MAA, is essential for early identification of women at risk for developing a poor MAA. Since the existing meta-analysis uses methods that only indirectly address those factors that are the most important determinants of MAA, this study provides an important contribution to the literature in this respect.

## Methods

### Study design

This is a prospective study comprising questionnaires and health records for pregnant women participating in antenatal care visits provided by their general practitioner (GP).

### Setting

The healthcare system in Denmark is taxpayer-funded and free of charge for the patient. The majority of Danes (99%) are registered with a GP who functions as a gatekeeper to secondary care. A minimum of three prenatal care visits are offered by the GP at pregnancy weeks 6–10, 25 and 32. A fourth postnatal visit is conducted eight weeks postpartum. Almost all women wanting to keep their pregnancy attend the first visit. In this consultation a structured record is established (the Pregnancy Health Record), which is then sent to midwives and the relevant obstetric services.

### Participants

192 randomly selected GP practices in The Capital Region and Region Zealand participated. A detailed description of the recruitment process is described elsewhere [26]. All women booking an appointment for a first antenatal care visit in the participating GPs’ practices between 1 April 2015 and 15 August 2016 were eligible for participation. All women were given oral and written information about the project at the first visit. If they decided to participate, they gave written consent granting access to the data from their pregnancy records and allowing the researchers to contact them and send questionnaires. Withdrawal of consent, miscarriage, induced abortion and stillbirth were exclusion criteria. All procedures were in accordance with the Helsinki II Declaration and Danish law. Approval from the Danish Data Protection Agency was obtained (Journal 2014–41-3018).

### Data

Data were collected from a clinical interview conducted by the GPs, reported in the Pregnancy Health Record as well as from electronic patient questionnaires. The Pregnancy Health Record is a two-page questionnaire that is initiated and completed by the GP at the first antenatal visit between gestational weeks 6–10. An electronic questionnaire developed for this study was sent by e-mail after each of the three antenatal care visits in pregnancy weeks 6–10, 25 and 32. Questionnaires were re-sent to non-responders after two weeks. If they still did not respond, then an e-mail and SMS were sent.

### Maternal antenatal attachment

To assess MAA we used the Maternal Antenatal Attachment Scale (MAAS) [4]. Compared to other existing measures of MAA (e.g. the Maternal Fetal Attachment Scale [5] or the Prenatal Attachment Inventory [6]) the MAAS is a relatively short questionnaire, making it more feasible to implement in clinical practice and studies with large questionnaire batteries like the current one. The scale has shown good psychometric properties in the original validation studies [4, 27]. As MAA generally increases with GA and the phenomenon is best captured late in pregnancy [9], the MAAS was included in the survey after the third antenatal visit.

The MAAS consists of 19 items each with five ordinal response options. Examples of items are: “Over the past two weeks when I think about the baby inside me I get feelings which are…” (answers ranging from “very sad” to “very happy”); “Over the past two weeks I have felt…” (answers ranging from “very emotionally distant from my baby” to “very close emotionally to my baby”). The questionnaire measures two dimensions of MAA: “quality” (11 items, henceforth called “Quality”) and “strength or intensity of preoccupation with the fetus” (8 items, henceforth called “Intensity”). Quality represents the mother’s emotional experiences towards the fetus (the conceptualization of the fetus as ‘a little person’, closeness, pleasure, and tenderness and distress at imagined loss versus distance and irritation). Intensity represents the intensity of preoccupation regarding time spent thinking about, talking to, and palpating the fetus, i.e. the extent to which the fetus occupies a central place in the woman’s emotional life. (Range for Quality: 11–50; range for Intensity: 8–40). A total MAAS score (henceforth “Global MAAS score”) can also be calculated and it is a composite of Intensity and Quality, (range: 19–95) with higher scores reflecting a stronger MAA.

For this study, the MAAS was translated into Danish in accordance with international standard translation procedures [28] including a pilot test on 15 pregnant women and adapted based on their feedback. This version was then back-translated into English blind to the original version of the MAAS. The Danish version of the MAAS showed acceptable to excellent levels of reliability: Cronbach’s alpha for the total score: = 0.81; for Quality = 0.72; and for Intensity = 0.73. These values are similar to the original validation study of the MAAS [4] and more recent validation studies [29, 30].

We used the two subscales Quality and Intensity as well as the Global MAAS score. For tabulation purposes, a binary variable indicating low versus normal MAA was used. Following Rowe et *al*., 2013 [31] we defined low MAA as a Global MAAS score ≤ 75.

The following factors measured in the first trimester (except major life events which was measured in the second trimester), were investigated as potential determinants of MAA.

Socio-demographic factors, Major life events [32, 33], Social network and support [34], Physical health, Mental health [35], Reproductive background and pregnancy related risk markers. Detailed definitions of the above factors are found in the Additional file 1: supplementary table, Additional file 2 and are described elsewhere [36].

### Statistical analysis

The purpose of the statistical analyses is to find out which items found in the pregnancy record or obtained through common clinical questions asked by the GP in the first pregnancy consultation yield most information about MAA. A dominance analysis that ranks the items for importance directly addresses this aim. The relative importance of the factors for the Global MAAS score and Quality and Intensity is calculated as the mean increase in the coefficient of determination, R^2^, associated with the addition of a factor to a multivariable linear regression model across all multivariable linear regression models that can be constructed from the remaining factors that were investigated. This procedure separates the R^2^ of the model with all factors included into parts attributable to each of the factors. The relative importance approach is discussed in the following references [41, 42]. Linear regression analysis for the five most important factors on each of the Global MAAS score and Quality and Intensity were used to characterise the size and direction of their effects. Analyses were done in R version 3.6.1*.*

## Results

**Fig. 1:**
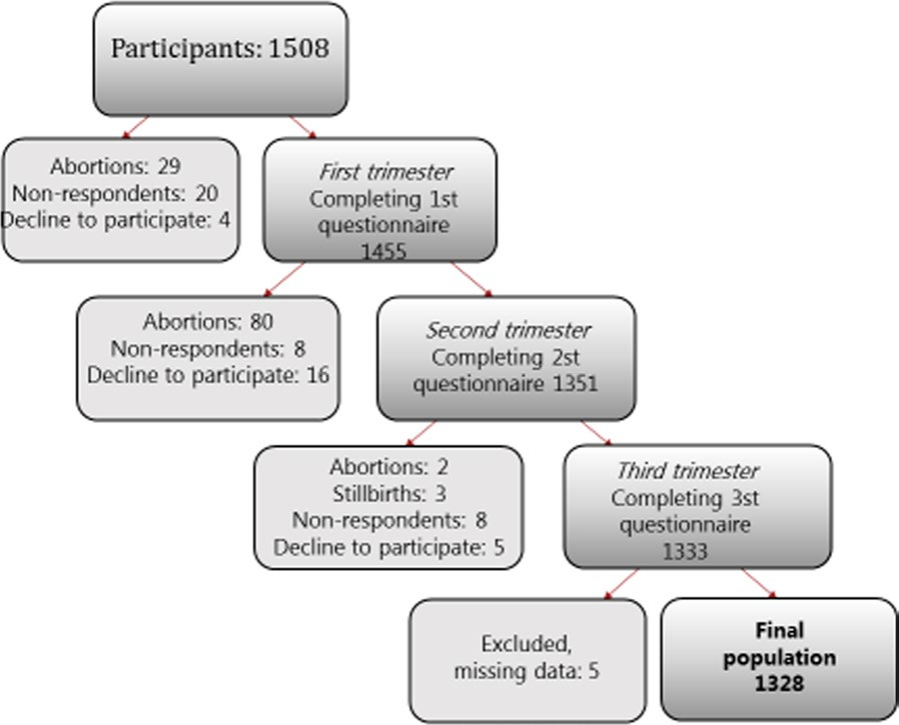
Flowchart of participants and drop-out

The flow of participants is shown in Fig. 1. A total of 1508 pregnant women were enrolled in the study. We received the third questionnaire from 1333 women (89%) and pregnancy health records from 1479 (98%). Both datasets were received for 1328 (88%), resulting in a final sample of 1328.

In the total sample, mean Global MAAS score was 77.4 (*SD* = 7.3), for Quality it was 45.7 (*SD* = 3.4), and for Intensity it was 27.6 (*SD* = 4.5). Low MAA (Global MAAS score ≤ 75) was observed for 513 (38.6%) women.

Women who score lower on the MAAS were older, had children living at home, higher income and largely reported not having access to practical help from family and friends when needed. Women who score lower on the MAAS were also more likely to be parous, to report self-rated health as fair and physical fitness as poor, have higher depression scores, a history of psychological difficulties, and lower WHO scores compared to women with normal MAA.

**Fig. 2:**
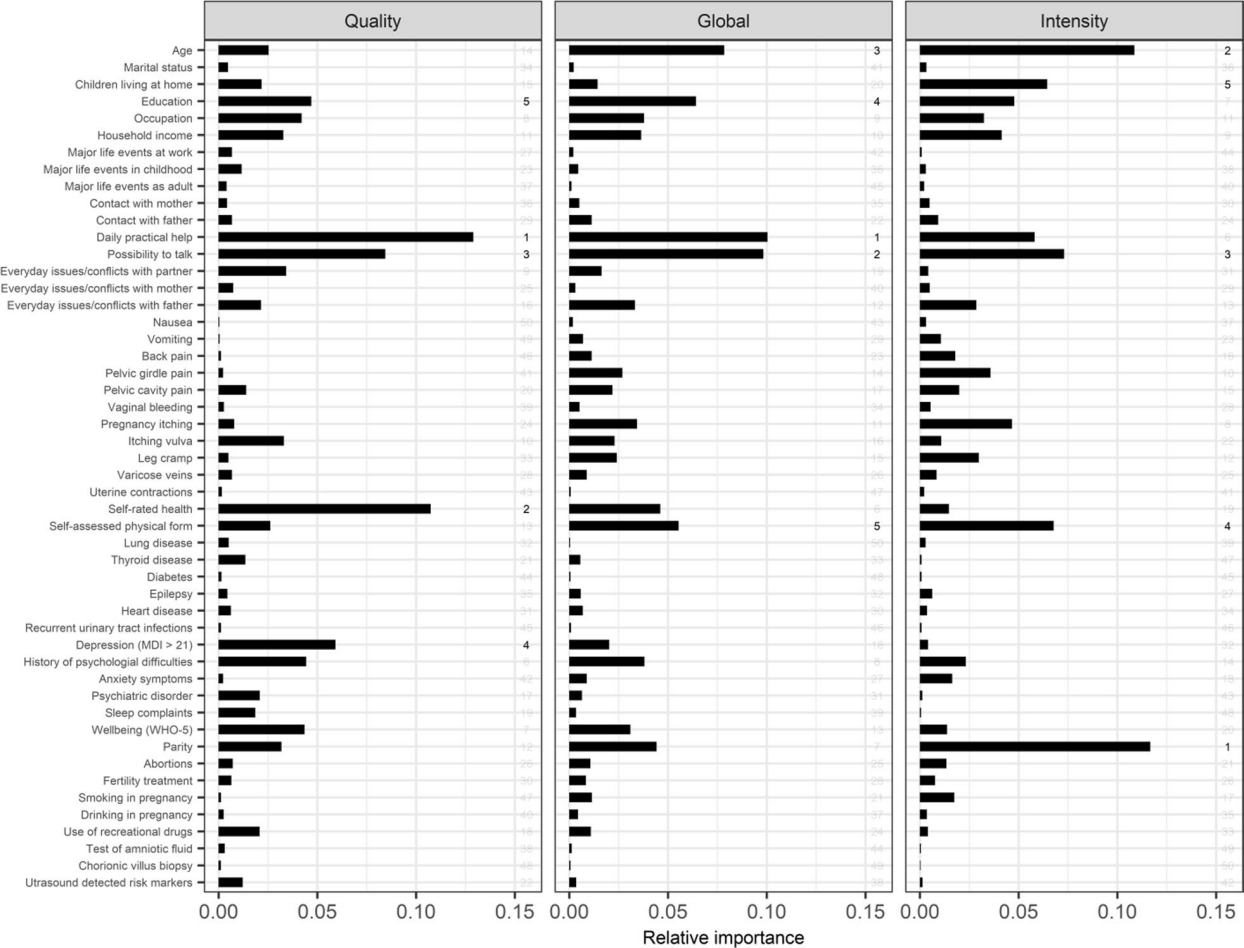
The relative importance in percentages of the R^2^ of the model with all factors included of the selection of the factors assessed for importance for Global MAAS and for the two subscales Quality and Intensity, respectively. Bigger bars indicate greater importance. In the right margin of each panel the top-five important factors are indicated

Figure 2 shows the relative importance of the full selection of factors for Quality, Intensity and the Global MAAS score. To facilitate interpretation, the five most important factors for each of the scales are indicated. The perceived social support variable ‘having someone to talk to when needed’ was among the five most important factors associated with both Quality and Intensity. Apart from this variable, different factors contributed to Quality and Intensity scores. For Quality, ‘lack of daily practical help’, self-rated health, depression, and level of education were the most important factors. For Intensity, parity, age, self-assessed physical fitness and having other children living at home were most important.

Table 1 reports the direction of the effects of the five most important factors for each of the three MAAS scales. The effect estimates show that MAAS scores decrease with increasing age, parity, increasing educational level, more symptoms of depression, worse self-rated health and poor physical fitness. Lack of daily practical help and not having someone to talk to when needed were also associated with lower scores on the MAAS.

**Table 1:** Multivariable linear regression analysis for the five most important factors on each of the Global MAAS score and Quality and Intensity subscales. The table shows the distribution of the factors, and the size and direction of their effects

	N (%)	Quality			Global			Intensity		
		Estimate	SE	*p *Value	Estimate	SE	*p *Value	Estimate	SE	*p *Value
*Age*										
< 26 years	160 (12.1)	–	–	–	Ref	Ref	Ref	Ref	Ref	Ref
26—30 years	466 (35.1)	–	–	–	− 1.29	0.65	0.05	− 1.04	0.40	0.01
31—35 years	454 (34.2)	–	–	–	− 2.54	0.68	0.00	− 2.29	0.42	0.00
36 + years	248 (18.7)	–	–	–	− 2.70	0.75	0.00	− 2.57	0.47	0.00
*Children living at home*										
No	537 (40.4)	–	–	–	–	–	–	Ref	Ref	Ref
Yes	791 (59.6)	–	–	–	–	–	–	0.33	0.56	0.55
*Education*										
ISCED Level 1–3	235 (17.7)	Ref	Ref	Ref	Ref	Ref	Ref	–	–	–
ISCED Level 4–5	433 (32.6)	− 0.05	0.26	0.83	0.62	0.57	0.28	–	–	–
ISCED Level 6	418 (31.5)	− 0.67	0.26	0.01	− 1.13	0.60	0.06	–	–	–
ISCED Level 7–8	242 (18.2)	− 1.15	0.29	0.00	− 2.59	0.69	0.00	–	–	–
*Daily practical help*										
0 relations	11 (0.8)	Ref	Ref	Ref	Ref	Ref	Ref	–	–	–
1 type of relationhip	29 (2.2)	4.12	1.11	0.00	5.72	2.43	0.02	–	–	–
2 types of relationship	87 (6.6)	3.25	1.01	0.00	4.33	2.20	0.05	–	–	–
3 types of relationship	153 (11.5)	2.57	0.99	0.01	3.24	2.16	0.13	–	–	–
4 types of relationship	256 (19.3)	3.63	0.98	0.00	5.58	2.14	0.01	–	–	–
5 types of relationship	484 (36.5)	3.49	0.97	0.00	4.81	2.12	0.02	–	–	–
6 types of relationship	308 (23.2)	3.74	0.99	0.00	6.24	2.15	0.00	–	–	–
*Opportunity to talk*										
1 type of relationship	2 (0.2)	Ref	Ref	Ref	Ref	Ref	Ref	Ref	Ref	Ref
2 types of relationship	41 (3.1)	2.97	2.28	0.19	8.91	4.98	0.07	4.49	3.11	0.15
3 types of relationship	92 (6.9)	3.64	2.24	0.11	10.65	4.90	0.03	5.16	3.07	0.09
4 types of relationship	221 (16.6)	3.82	2.23	0.09	11.24	4.87	0.02	5.92	3.05	0.05
5 types of relationship	456 (34.3)	3.88	2.23	0.08	11.13	4.86	0.02	5.49	3.04	0.07
6 types of relationship	516 (38.9)	4.11	2.23	0.07	11.47	4.87	0.02	6.03	3.04	0.05
*Self-rated health (SRH)*										
Very good	190 (14.3)	Ref	Ref	Ref	–	–	–	–	–	–
Good	858 (64.6)	− 0.71	0.25	0.01	–	–	–	–	–	–
Fair	251 (18.9)	− 1.34	0.31	0.00	–	–	–	–	–	–
Poor	29 (2.2)	− 0.26	0.64	0.68	–	–	–	–	–	–
*Self-assessed physical fitness (SAPF)*										
Very good	42 (3.2)	–	–	–	Ref	Ref	Ref	Ref	Ref	Ref
Good	341 (25.7)	–	–	–	− 2.45	1.12	0.03	− 1.83	0.71	0.01
Fair	625 (47.1)	–	–	–	− 2.63	1.10	0.02	− 1.88	0.69	0.01
Poor	290 (21.8)	–	–	–	− 3.99	1.14	0.00	− 2.71	0.72	0.00
Very poor	30 (2.3)	–	–	–	− 6.31	1.66	0.00	− 2.75	1.04	0.01
*Depression (MDI > 21)*										
No	1096 (82.5)	Ref	Ref	Ref	–	–	–	–	–	–
Yes	232 (17.5)	− 0.97	0.24	0.00	–	–	–	–	–	–
Parity										
No previous births	589 (44.4)	–	–	–	–	–	–	Ref	Ref	Ref
One previous births	502 (37.8)	–	–	–	–	–	–	− 2.08	0.57	0.00
Several previous births	237 (17.9)	–	–	–	–	–	–	− 1.29	0.62	0.04

## Discussion

While previous studies on determinants of MAA have mainly focused on socioeconomic status, psychosocial risk factors and mental health, including depression, anxiety and perceived social support [9], the present study adds to the literature by including physical health and discomfort. Specifically and pragmatically, we investigated the relative importance of factors from the first trimester which are either found in the woman’s pregnancy record or are easily obtained during a first pregnancy consultation to the development of MAA measured in the third trimester when it is expected to be strongest [9].

We found that self-rated health early in pregnancy is the second most important determinant of the Quality of MAA. Higher scores predict more feelings of closeness and tenderness towards the fetus. Along the same lines, perceived physical fitness emerged as one of the most important factors for the Intensity of MAA, with higher scores predicting more time spent thinking about, talking to, and feeling for the fetus. As no previous study has investigated such factors as predictors of MAA, we cannot directly compare these results with others. However, one study [37] reports that self-reported well-being during the third trimester is positively associated with the Quality of MAA. Another study reports that exercise during pregnancy improved maternal/fetus interaction significantly, reduced discomfort, and helped pregnant women to maintain physical well-being [38]. These results suggest that a general feeling of being healthy and physical wellness play a role in how the expectant woman adapts to pregnancy, her successful transition to motherhood, and subsequently in the development of feeling connected with the fetus. That said, it is possible that physical health and mental wellbeing interact and buffer or magnify the effects of the other; for example, poor mental health may increase the adverse effects of poor physical health or mental wellbeing may buffers the negative effects of poor physical health. This question is nevertheless outside the scope of the present study, and should be addressed in future research.

Confirming results from the existing meta-analysis [9] perceived social support (‘having someone to talk to when needed’ and ‘lack of practical help’) appeared to be associated with the development of MAA. Specifically, the variable ‘having someone to talk to when needed’, was an important determinant of Quality, Intensity and the Global MAAS score. Within the attachment literature, perceived social support and help seeking behaviour is often linked with adult attachment security (e.g. [39]). The social support questions used in this study may reflect aspects of the expectant mother’s own attachment orientation—which in turn probably plays a role in MAA. Indeed, adult attachment security has been found to be associated with how individuals perceive the availability and trustworthiness of others; and perceptions of social support (i.e. the availability and quality of assistance provided by others) are more likely to be distorted in insecure adults compared with secure adults [40]. Furthermore, insecurely attached adults more often perceive social support to be less available to them [41] and they may tend to interpret the provided support in a more negative way [42]. Support for the notion that the expectant mother’s own attachment security explains the strong association between perceived social support in the first trimester and MAA in our study, comes from a study reporting that adult secure attachment was positively correlated with scores on the Quality scale of MAAS and insecure dismissing attachment was negatively correlated with the Global MAAS score when measured concurrently [29].

Consistent with the existing literature [9, 13, 14, 37] symptoms of depression in the first trimester are important indicators of the Quality of MAA. Considering the high levels of comorbidity in psychiatric conditions, and in particular in perinatal mental disorders (see for example [43]), it is somewhat surprising that neither anxiety nor having a psychiatric diagnosis are of high importance for MAA in our study. However, one study reports results consistent with ours; stress, anxiety and adverse life events are not strongly related to MAA [44].

Finally, our results show that age and having one or more children before this pregnancy are important early determinants of the intensity of MAA. In line with previous studies using the MAAS [8, 29, 45, 46], we find that older women and women with more children tend to be less preoccupied with the fetus, i.e. score lower on the MAAS Intensity scale. A possible explanation could be that women who already have one or more children are less focused on the fetus, compared with primiparous women, as the transition to motherhood [1] happened during the first pregnancy. Moreover, we find level of education to be an important determinant of the Quality of MAA, with higher educational level predicting scoring lower on the MAAS Quality scale. This result is consistent with a previous study reporting that women without a degree had higher scores on the total MAAS compared to women with a higher education [30]. Other studies [29, 47] report that more highly educated women score lower on the counterpart of the MAAS, the Maternal Postpartum Attachment Scale, and similar findings were reported by Reck et al., in postpartum women [48] and Goecke et al., in pregnant women [37]. As suggested by Reck and colleagues, women with a higher education may generally answer in a more honest and less socially accepted manner [48], and it could be argued that increasing age should per se not be considered a risk factor for the emergent relationship with the offspring. An explanation for these findings taken together could be that more highly educated women, women with other children, and older women attribute less value to motherhood in terms of role fulfilment and are more realistic and concerned about those changes that pregnancy imposes on the body and that come with parenthood [29, 30, 45].

Two reviews [23, 24] have previously reported findings on the relationships between MAA and psychosocial, pregnancy-related, and health factors. Our study adds to this literature by addressing physical pregnancy-related symptoms. Furthermore, our method of analysis more directly addresses the relative importance of the factors. Our study sample responds to the reviews’ call for large and diverse samples. There is ample opportunity in forthcoming reports to assess the relationships between MAA and aspects of prenatal care throughout pregnancy, and identify the association of MAA with relevant maternal/infant outcomes, such as infant development*.*

### Strengths and weaknesses of the study

To date, the present study using a sample of 1328 women is the largest single study of predictors of maternal antenatal attachment [9, 29]. While previous studies have used convenience samples, our study is based on a sequential and near-complete recruitment and retention over a 4–5 month period of the pregnant women on the list of a random selection of Danish GPs.

We used no exclusion criteria for the recruitment. Furthermore, there were very few non-responders, and complete data were obtained from almost all invited women. Not all eligible women were invited to participate and it appears that the GPs may have avoided approaching women who did not speak Danish [36]*.* Many of these women would not be able to complete the Danish MAAS and it is possible that for these women, maybe because of cultural background, MAA may have different drivers. Hence, the presented results may not generalize to this subpopulation, but can be considered representative of the great majority of pregnant women in Denmark.

We deliberately used an analysis approach that directly addresses the relative importance of a set of possible predictive factors. While this approach is atheoretical in that it does not attempt to infer causal relationships, it focusses the inquiry of the GP towards assessing the risk of low attachment. This is also why we pragmatically limited the set of factors investigated to those found in the pregnancy record of the women, along with some clinical questions easily asked by the GP. Consequently, the mode of inquiry may be affected by the GP’s questioning style or the woman’s reporting style, but this is the way most of the information about the pregnant woman is obtained by the GP.

Hence, this study provides a significant contribution to the field. We employed an analytical approach where the relative importance of a set of potential determinants is assessed directly. Traditional attempts to assess relative importance indirectly by model selection techniques may miss important factors, particularly when these factors are correlated.

### Conclusions

Maternal general mental and physical well-being together with perceived social support in the first trimester are important indicators of how the pregnant mother may develop MAA. In terms of clinical implications, our findings suggest that an approach targeting both psychosocial and physiological well-being may positively influence MAA, supporting pregnant women to do what they can to increase overall well-being, e.g. through exercise [44, 49] or through psychotherapy focusing on improving close relationships and the emergent relationship with the child [50]. Special attention should be paid to women who do not feel healthy, who lack social support, and who present depressive symptoms. These characteristics are risk factors for the mother’s successful adaptation to motherhood and for her emergent relationship with the unborn child, and point to the potential benefits of early supportive interventions.

## Supplementary information

**Additional file 1: Table 1**. Characteristics of the pregnant women.

**Additional file 2**. Supplementary file.

## Data Availability

The datasets used and/or analysed during the present study are available from the corresponding author on reasonable request.

## Abbreviations

MAA: Maternal antenatal attachment
MAAS: Maternal antenatal attachment scale
GPs: General practitioners
MDI: Major depression inventory
ASS: Anxiety symptom scale
WHO-5: The World Health Organisation-five well-being index

## References

[1] Slade A (2009). Review of attachment in psychotherapy. Psychotherapy (Chicago, Ill).

[2] Pisoni C, Garofoli F, Tzialla C, Orcesi S, Spinillo A, Politi P (2016). Complexity of parental prenatal attachment during pregnancy at risk for preterm delivery. J Maternal Fetal Neonatal Med.

[3] Slade A CL, Sadler LS, Miller M, 3:22–39. 2009, 3:22–39. The psychology and psychopathology of pregnancy. Handbook of infant mental health 2009.

[4] Condon JT (1993). The assessment of antenatal emotional attachment: development of a questionnaire instrument. Br J Med Psychol.

[5] Cranley MS. Development of a tool for the measurement of maternal attachment during pregnancy. Nursing research. 1981. 6912989

[6] Muller ME, Mercer RT (1993). Development of the prenatal attachment inventory. West J Nurs Res.

[7] Craley M (1981). Development of a tool for the measurement of maternal attachment during pregnancy. Nursing. Res.

[8] Salisbury A, Law K, LaGasse L, Lester B (2003). Maternal-fetal attachment. JAMA.

[9] Yarcheski A, Mahon NE, Yarcheski TJ, Hanks MM, Cannella BL (2009). A meta-analytic study of predictors of maternal-fetal attachment. Int J Nurs Stud.

[10] Cassidy J. The nature of the child's ties. 2008.

[11] Hesse E. The Adult Attachment Interview: Protocol, method of analysis, and empirical studies. 2008.

[12] Van den Bergh B, Simons A (2009). A review of scales to measure the mother-foetus relationship. J Reprod Infant Psychol..

[13] Rossen L, Hutchinson D, Wilson J, Burns L, C AO, Allsop S, et al. Predictors of postnatal mother-infant bonding: the role of antenatal bonding, maternal substance use and mental health. Archives of Women's Mental Health. 2016;19(4):609–22. 10.1007/s00737-016-0602-z 26867547

[14] Petri E, Palagini L, Bacci O, Borri C, Teristi V, Corezzi C (2018). Maternal-foetal attachment independently predicts the quality of maternal-infant bonding and post-partum psychopathology. J Maternal Fetal Neonatal Med.

[15] McMahon CCA, Berry S, Gibson F (2016). Maternal mind-mindedness: relations with maternal–fetal attachment and stability in the first two years of life: findings from an Australian prospective study. Infant Mental Health J.

[16] Foley S, Hughes C (2018). Great expectations? Do mothers’ and fathers’ prenatal thoughts and feelings about the infant predict parent-infant interaction quality? A meta-analytic review. Dev Rev.

[17] Verhage ML, Schuengel C, Madigan S, Fearon R, Oosterman M, Cassibba R (2016). Narrowing the transmission gap: a synthesis of three decades of research on intergenerational transmission of attachment. Psychol Bull.

[18] Groh AM, Roisman GI, van Ijzendoorn MH, Bakermans-Kranenburg MJ, Fearon RP (2012). The significance of insecure and disorganized attachment for children's internalizing symptoms: a meta-analytic study. Child Dev.

[19] Fearon RP, Bakermans-Kranenburg MJ, van Ijzendoorn MH, Lapsley AM, Roisman GI (2010). The significance of insecure attachment and disorganization in the development of children's externalizing behavior: a meta-analytic study. Child Dev.

[20] Groh AM, Fearon RP, Bakermans-Kranenburg MJ, van Ijzendoorn MH, Steele RD, Roisman GI (2014). The significance of attachment security for children's social competence with peers: a meta-analytic study. Attach Hum Dev.

[21] Kingston D, Heaman M, Fell D, Chalmers B (2012). Comparison of adolescent, young adult, and adult women's maternity experiences and practices. Pediatrics.

[22] Goodman SH, Rouse MH, Connell AM, Broth MR, Hall CM, Heyward D (2011). Maternal depression and child psychopathology: a meta-analytic review. Clin Child Fam Psychol Rev.

[23] Alhusen JL (2008). A literature update on maternal-fetal attachment. J Obstet Gynecol Neonatal Nurs.

[24] Cannella BL (2005). Maternal–fetal attachment: an integrative review. J Adv Nurs.

[25] Glynn LM, Howland MA, Fox M (2018). Maternal programming: Application of a developmental psychopathology perspective. Dev Psychopathol.

[26] Ertmann RK, Nicolaisdottir DR, Kragstrup J, Siersma V, Lutterodt MC (2020). Sleep complaints in early pregnancy A cross-sectional study among women attending prenatal care in general practice. BMC Pregnancy Childbirth..

[27] Condon JT, Corkindale C (1997). The correlates of antenatal attachment in pregnant women. Br J Med Psychol.

[28] Dewolf LKM, Velikova G, Johnson C, Scott N, Bottomley A, Group EQoL. EORTC Quality of Life Group translation procedure. 2009.

[29] van Bussel JC, Spitz B, Demyttenaere K (2010). Three self-report questionnaires of the early mother-to-infant bond: reliability and validity of the Dutch version of the MPAS, PBQ and MIBS. Arch Women's Mental Health.

[30] van Bussel JC, Spitz B, Demyttenaere K (2010). Reliability and validity of the Dutch version of the maternal antenatal attachment scale. Arch Women's Mental Health.

[31] Wynter K, Rowe H, Fisher J (2013). Common mental disorders in women and men in the first six months after the birth of their first infant: a community study in Victoria. Aust J Affect Disord.

[32] Holmes THRR (1967). The social readjustments rating scale. J Psychosom Res..

[33] Andersen I, Diderichsen F, Kornerup H, Prescott E, Rod NH (2011). Major life events and the risk of ischaemic heart disease: does accumulation increase the risk?. Int J Epidemiol.

[34] Lund R, Nielsen LS, Henriksen PW, Schmidt L, Avlund K, Christensen U (2014). Content validity and reliability of the Copenhagen social relations questionnaire. J Aging Health.

[35] Bech P (2012). Clinical psychometrics.

[36] Ertmann RK, Nicolaisdottir DR, Kragstrup J, Siersma V, Lutterodt MC, Bech P (2019). Physical discomfort in early pregnancy and postpartum depressive symptoms. Nord J Psychiatr.

[37] Goecke TW, Voigt F, Faschingbauer F, Spangler G, Beckmann MW, Beetz A (2012). The association of prenatal attachment and perinatal factors with pre- and postpartum depression in first-time mothers. Arch Gynecol Obstet.

[38] Ji ES, Han HR (2010). The effects of Qi exercise on maternal/fetal interaction and maternal well-being during pregnancy. J Obstetr Gynecol Neonatal Nurs.

[39] Magai C. Attachment in middle and later life2008.

[40] Rowe HJ WK, Steele A, Fisher JR, Quinlivan JA. The growth of maternal-fetal emotional attachment in pregnant adolescents: a prospective cohort study. J Pediatr Adolesc Gynecol 2003;10(1). 10.1016/j.jpag.2013.06.009 24075091

[41] Meredith P, Ownsworth T, Strong J (2008). A review of the evidence linking adult attachment theory and chronic pain: presenting a conceptual model. Clin Psychol Rev.

[42] Collins NL, Feeney BC (2004). Working models of attachment shape perceptions of social support: evidence from experimental and observational studies. J Pers Soc Psychol.

[43] Falah-Hassani K, Shiri R, Dennis CL (2017). The prevalence of antenatal and postnatal co-morbid anxiety and depression: a meta-analysis. Psychol Med.

[44] Rusanen E, Lahikainen AR, Polkki P, Saarenpaa-Heikkila O, Paavonen EJ (2018). The significance of supportive and undermining elements in the maternal representations of an unborn baby. J Reprod Infant Psychol.

[45] Damato EG (2004). Predictors of prenatal attachment in mothers of twins. J Obstetr Gynecol Neonatal Nurs JOGNN.

[46] Hjelmstedt A, Widstrom AM, Collins A (2006). Psychological correlates of prenatal attachment in women who conceived after in vitro fertilization and women who conceived naturally. Birth (Berkeley, Calif).

[47] Condon JT CC. The assessment of parent-to-infant attachment: Development of a self-report questionnaire instrument. J Reprod Infant Psychol. 1998;16(1).

[48] Reck C, Klier CM, Pabst K, Stehle E, Steffenelli U, Struben K (2006). The German version of the Postpartum Bonding Instrument: psychometric properties and association with postpartum depression. Arch Women's Mental Health.

[49] Martinsen EW (2008). Physical activity in the prevention and treatment of anxiety and depression. Nord J Psychiatry.

[50] Markin RD (2013). Mentalization-based psychotherapy interventions with mothers-to-be. Psychotherapy (Chicago, Ill).

[51] Topp CW, Ostergaard SD, Sondergaard S, Bech P (2015). The WHO-5 Well-Being Index: a systematic review of the literature. Psychother Psychosom.

