# Supplementary material for: What factors are most important for the development of the maternal–fetal relationship? A prospective study among pregnant women in Danish general practice
Source: BMC Psychol. 2021 Jan 4;9:2. doi: 10.1186/s40359-020-00499-x (PMC7784374; doi:10.1186/s40359-020-00499-x)
Supplement: Supplementary file 1 — Additional file 1: Table 1. Characteristics of the pregnant women. [file 40359_2020_499_MOESM1_ESM.docx]

Supplementary file: Table

Table: Characteristics of the pregnant women

|  | Total (n=1328) | |
| --- | --- | --- |
|  | n | % |
| **Socio-demographic** |  |  |
| **Age** |  |  |
| < 26 years | 160 | 12.1 |
| 26-30 years | 466 | 35.1 |
| 31-35 years | 454 | 34.2 |
| 36+ years | 248 | 18.7 |
| **Marital status** |  |  |
| Married or cohabiting | 1261 | 95.0 |
| Other | 15 | 1.1 |
| Alone | 52 | 3.9 |
| **Children living at home** |  |  |
| No | 537 | 40.4 |
| Yes | 791 | 59.6 |
| **Education** |  |  |
| ISCED Level 1-3 (lower secondary or less) | 235 | 17.7 |
| ISCED Level 4-5 (post secondary + short-cycle tertiary) | 433 | 32.6 |
| ISCED Level 6 (bachelor or equivalent) | 418 | 31.5 |
| ISCED Level 7-8 (master + doctor or equivalent ) | 242 | 18.2 |
| **Occupation** |  |  |
| Employed | 1001 | 75.4 |
| Unemployed | 70 | 5.3 |
| Student | 180 | 13.6 |
| Other | 52 | 3.9 |
| Sick leave | 25 | 1.9 |
| **Household income** |  |  |
| ≤ 299,999 kr | 160 | 12.1 |
| 300,000-599,999 kr | 400 | 30.1 |
| 600,000-899,999 kr | 448 | 33.7 |
| ≥ 900,000 kr | 140 | 10.5 |
| Unwilling to answer | 180 | 13.6 |
| **Smoking in pregnancy** |  |  |
| No | 1239 | 93.3 |
| Yes | 89 | 6.7 |
| **Drinking in pregnancy** |  |  |
| No | 1318 | 99.3 |
| Yes | 10 | 0.8 |
| **Use of recreational drugs in pregnancy** |  |  |
| No | 1324 | 99.7 |
| Yes | 4 | 0.3 |
| **Major life events** |  |  |
| **Major life events at work** |  |  |
| No events | 836 | 63.0 |
| One event | 335 | 25.2 |
| More events | 157 | 11.8 |
| **Major life events in childhood** |  |  |
| No events | 782 | 58.9 |
| One event | 284 | 21.4 |
| More events | 262 | 19.7 |
| **Major life events as adult** |  |  |
| No events | 612 | 46.1 |
| One event | 545 | 41.0 |
| More events | 171 | 12.9 |
| **Social network and support** |  |  |
| **Contact with mother** |  |  |
| Often | 1210 | 91.1 |
| Rarely | 50 | 3.8 |
| Mother absent | 68 | 5.1 |
| **Contact with father** |  |  |
| Often | 962 | 72.4 |
| Rarely | 215 | 16.2 |
| Father absent | 151 | 11.4 |
| **Daily practical help from family and friends (Number of different types of relationships out of six)** |  |  |
| No one | 11 | 0.8 |
| 1 person | 29 | 2.2 |
| 2 persons | 87 | 6.6 |
| 3 persons | 153 | 11.5 |
| 4 persons | 256 | 19.3 |
| 5 persons | 484 | 36.5 |
| 6 persons | 308 | 23.2 |
| **Possibility to talk with family and friends (Number of different types of relationships out of six)** |  |  |
| No one | 0 | 0.0 |
| 1 person | 2 | 0.2 |
| 2 persons | 41 | 3.1 |
| 3 persons | 92 | 6.9 |
| 4 persons | 221 | 16.6 |
| 5 persons | 456 | 34.3 |
| 6 persons | 516 | 38.9 |
| **Everyday issues with partner** |  |  |
| Partner absent | 28 | 2.1 |
| No issues | 1184 | 89.2 |
| One issue | 75 | 5.6 |
| Several issues | 41 | 3.1 |
| **Everyday issues with mother** |  |  |
| Mother absent | 69 | 5.2 |
| No issues | 1147 | 86.4 |
| One issue | 79 | 6.0 |
| Several issues | 33 | 2.5 |
| **Everyday issues with farther** |  |  |
| Father absent | 151 | 11.4 |
| No issues | 1107 | 83.4 |
| One issue | 48 | 3.6 |
| Several issues | 22 | 1.7 |
| **Physical health** |  |  |
| **Pregnancy-related symptoms** |  |  |
| **Nausea** |  |  |
| No | 147 | 11.1 |
| Yes | 1181 | 88.9 |
| **Vomiting** |  |  |
| No | 789 | 59.4 |
| Yes | 539 | 40.6 |
| **Back pain** |  |  |
| No | 835 | 62.9 |
| Yes | 493 | 37.1 |
| **Pelvic girdle pain** |  |  |
| No | 582 | 43.8 |
| Yes | 746 | 56.2 |
| **Pelvic cavity pain** |  |  |
| No | 886 | 66.7 |
| Yes | 442 | 33.3 |
| **Vaginal bleeding** |  |  |
| No | 1106 | 83.3 |
| Yes | 222 | 16.7 |
| **Pragnancy itching** |  |  |
| No | 1107 | 83.4 |
| Yes | 221 | 16.6 |
| **Itching vulva** |  |  |
| No | 1075 | 81.0 |
| Yes | 253 | 19.1 |
| **Leg cramp** |  |  |
| No | 1195 | 90.0 |
| Yes | 133 | 10.0 |
| **Varicose veins** |  |  |
| No | 1293 | 97.4 |
| Yes | 35 | 2.6 |
| **Uterine contractions** |  |  |
| No | 1266 | 95.3 |
| Yes | 62 | 4.7 |
| **Self-rated health (SRH)** |  |  |
| Very good | 190 | 14.3 |
| Good | 858 | 64.6 |
| Fair | 251 | 18.9 |
| Poor | 29 | 2.2 |
| Very poor | 0 | 0.0 |
| **Self-assessed physical form (SAPF)** |  |  |
| Very good | 42 | 3.2 |
| Good | 341 | 25.7 |
| Fair | 625 | 47.1 |
| Poor | 290 | 21.8 |
| Very poor | 30 | 2.3 |
| **Lung disease** |  |  |
| No | 1233 | 92.9 |
| Yes | 95 | 7.2 |
| **Thyroid disease** |  |  |
| No | 1277 | 96.2 |
| Yes | 51 | 3.8 |
| **Diabetes** |  |  |
| No | 1318 | 99.3 |
| Yes | 10 | 0.8 |
| **Epilepsy** |  |  |
| No | 1314 | 99.0 |
| Yes | 14 | 1.1 |
| **Heart disease** |  |  |
| No | 1270 | 95.6 |
| Yes | 58 | 4.4 |
| **Recurrent urinary tract infections** |  |  |
| No | 1268 | 95.5 |
| Yes | 60 | 4.5 |
| **Mental health** |  |  |
| **Depression symptoms (depression score > 21)** |  |  |
| No | 1096 | 82.5 |
| Yes | 232 | 17.5 |
| **History of psychological difficulties** |  |  |
| No | 672 | 50.6 |
| Yes but I did not seek treatment | 257 | 19.4 |
| Yes, and I did seek treatment | 399 | 30.1 |
| **Anxiety symptoms (anxiety score > 10)** |  |  |
| No | 1233 | 92.9 |
| Yes | 95 | 7.2 |
| **Psychiatric disorder** |  |  |
| No | 1233 | 92.9 |
| Yes | 95 | 7.2 |
| **Sleep complaints** |  |  |
| No | 1050 | 79.1 |
| Yes | 278 | 20.9 |
| **Wellbeing (WHO-5)** |  |  |
| WHO-5 score < 70 | 296 | 22.3 |
| WHO-5 score ≥ 70 | 1032 | 77.7 |
| **Reproductive background and risk** |  |  |
| **Amnioticentesis** |  |  |
| Yes | 47 | 3.5 |
| No | 1281 | 96.5 |
| **Chorionic villus biopsy** |  |  |
| Yes | 47 | 3.5 |
| No | 1281 | 96.5 |
| **Ultrasound dectected risk markers** |  |  |
| No scan done | 201 | 15.1 |
| Scan done and there were no risk markers present | 30 | 2.3 |
| Scan done and there risk markers were unclear | 1082 | 81.5 |
| Scan done and there were risk markers present | 15 | 1.1 |
| **Fertility treatment** |  |  |
| No | 1197 | 90.1 |
| Yes | 131 | 9.9 |
| **Parity** |  |  |
| No previous births | 589 | 44.4 |
| One previous birth | 502 | 37.8 |
| Several previous births | 237 | 17.9 |
| **Abortions** |  |  |
| No | 837 | 63.0 |
| One, spontaneous | 172 | 13.0 |
| Several, last spontaneous | 98 | 7.4 |
| One, not spontaneous | 170 | 12.8 |
| Several, last not spontaneous | 51 | 3.8 |
