# Supplementary material for: What factors are most important for the development of the maternal–fetal relationship? A prospective study among pregnant women in Danish general practice
Source: BMC Psychol. 2021 Jan 4;9:2. doi: 10.1186/s40359-020-00499-x (PMC7784374; doi:10.1186/s40359-020-00499-x)
Supplement: Supplementary file 2 — Additional file 2. Supplementary file. [file 40359_2020_499_MOESM2_ESM.docx]

**Supplementary file**

*Socio-demographic factors*: Age (< 25 years, 26-30 years, 31-35 years, 36+ years), marital status (married or cohabiting, lone parent), children living at home (no/yes), years of education (0-10 years, 11-13 years, 14-16 years, >16 years), years of completed or on-going education (International Standard Classification of Education (ISCED). Level 1-3 = 3, 4 and 5, 6, 7 and 8), occupation (employed, unemployed, student, other, sick leave), income of household (<39,999 EUR, 40,000-79,999 EUR, 80,000-119,999 EUR, ≥120,000EUR, do not want to answer). Major life events: number of major life events at work, in childhood and in adult life (0, 1 or 2+, for seven items). These questions were based on a shorter and modified version of the Social Readjustments Rating Scale [35, 36].

*Social network and support*: Contact with mother/father (often, rarely, do not have any), daily help from family and friends (number of different types of relationship out of six), opportunity to talk with family and friends if needed (number of different types of relationships out of six), experiences of everyday issues/conflicts with partner, mother or father (do not have a mother/father/partner, do have a mother/farther/partner and no problem, do have a mother/father/partner and one problem, do have a mother/father/partner and two or more problems) [37].

*Physical health*: Specific pregnancy-related symptoms: nausea, vomiting, back pain, pelvic girdle pain, pelvic cavity pain, itching of vulva, varicose veins, leg cramps, pregnancy itching, vaginal bleeding and uterine contractions (no, yes), self-rated health (very good, good, poor, very poor), self-assessed fitness (very good, good, poor, very poor), chronic diseases: heart disease, lung disease, thyroid disease, diabetes, epilepsy, recurrent urinary tract infections (yes/no).

*Mental health:* Previous psychological difficulties (no – yes /but did not seek professional health - yes/ seek professional health), symptoms of depression (MDI >20) [38] anxiety (ASS >10) [38], known psychiatric disorder (yes/no), sleep complaints (yes/no), wellbeing (WHO-5 < 70)[39]. Details about MDI and ASS have been described elsewhere [40].

*Reproductive background and pregnancy related risk markers:* Parity (0, 1, >1 children), abortions (0, 1, >1 abortions), in vitro fertilization (yes/no). Smoking, drinking or use of recreational drugs during pregnancy (yes/no). Amniotic fluid sampling (yes/no), chorionic villus biopsy (yes/no), ultrasound detected risk markers/malformations in the child (1,2 or 3 risk markers).
